# The Impact of Diabetes Mellitus on Renal Cell Carcinoma Prognosis: A Meta-Analysis of Cohort Studies

**DOI:** 10.1097/MD.0000000000001055

**Published:** 2015-07-02

**Authors:** Luyao Chen, Hongzhao Li, Liangyou Gu, Xin Ma, Xintao Li, Yu Gao, Yu Zhang, Donglai Shen, Yang Fan, Baojun Wang, Xu Bao, Xu Zhang

**Affiliations:** From the State Key Laboratory of Kidney Diseases, Department of Urology, Chinese PLA Medical School, Chinese PLA General Hospital, Beijing (LC, HL, LG, XM, XL, YG, YZ, DS, YF, BW, XZ); and Medical School, Nankai University, Tianjin, People's Republic of China (XB).

## Abstract

Previous studies that investigated the relationship between DM and survival in renal cell carcinoma (RCC) patients reported inconsistent findings. Hence, we conducted a meta-analysis to obtain a more precise evaluation of the prognostic significance of DM in RCC. A systematic review was conducted with PubMed, Embase, and Web of Science to identify relevant articles that evaluated the effect of DM on RCC patients. Based on the inclusion and quality assessment criteria, 18 studies were eligible for the meta-analysis. Pooled hazard ratios (HR) and corresponding 95% confidence intervals (CI) for overall survival (OS), cancer-specific survival (CSS), and recurrence-free survival (RFS) were calculated by standard meta-analysis techniques. The results suggested that DM was associated with poor OS (HR 1.56, 95% CI, 1.35–1.81, *P* < 0.001), poor CSS (HR 2.03, 95% CI, 1.37–3.01, *P* < 0.001), and poor RFS (HR 1.73, 95% CI, 1.25–2.39, *P* = 0.012). In addition, for patients with localized RCC, patients with clear cell RCC, or patients receiving nephrectomy, DM was associated with both poor OS and CSS by subgroup analyses. Our study revealed that there was a significant negative impact of DM on OS, CSS, and RFS in RCC patients. Therefore, more attention should be paid to RCC patients with preexisting DM because of their poor prognosis.

## INTRODUCTION

Renal cell carcinoma (RCC) is the third most common malignancy in the urogenital system, with approximately 25% of patients found to have metastases at first diagnosis.^1^ The incidence of RCC has steadily increased in the last 4 decades, accounting for 2%–3% of human cancers.^2^ The dismal prevalence of RCC prompts the need for outcome prediction models that can be used in counseling patients, selecting individualized treatment, and making surveillance programs especially after surgery. Currently, the tumor, nodes, metastasis (TNM) staging system are still regarded as one of the most important RCC prognostic factors. Thus, multiple RCC prognostic models have been constructed that primarily concentrate on TNM stage, nuclear grade, and performance status. Recently, a considerable number of research studies have focused on relevant metabolic factors affecting the prognosis of RCC; these studies serve as an additional guide in decision-making for therapeutic strategies to improve prognosis.^3–5^

Diabetes mellitus (DM) is one of the most common metabolic diseases. Nearly 285 million people worldwide suffer from DM in 2010, and the global prevalence of this chronic disorder is increasing rapidly.^6^ DM has a tremendous effect on human health and is considered a well-known cause of cardiovascular complications, including stroke, coronary heart disease, renal disease, and neuropathy.^7^ More recently, DM was reported to be associated with increased incidence and elevated risk of mortality in numerous cancers, such as liver, prostatic, and endometrial cancers.^8–10^ However, findings of previous studies that investigated the relationship between DM and survival in RCC patients were inconsistent. Psutka et al^11^ found that DM was independently associated with decreased cancer-specific survival (CSS) and overall survival (OS) in patients with surgically treated clear cell RCC. Lee et al^12^ also demonstrated that DM was a prognostic factor predicting worse OS in RCC patients. However, Hofner et al^13^ suggested that preexisting DM had no significant effect on the outcome of localized RCC.

To obtain a more precise evaluation of the prognostic significance of DM in RCC patients, we conducted a systematic review of published studies and carried out a standard meta-analysis of extracted data that can be merged.

## METHODS

### Literature Search Strategy

This meta-analysis was conducted according to the checklist of Meta-Analysis of Observational Studies in Epidemiology (MOOSE),^14^ and the Preferred Reporting Items for Systematic Reviews and Meta-Analyses (PRISMA).^15^

We searched 3 electronic databases, namely PubMed, Embase, and Web of Science, from their inception to February 2015. Search terms using MeSH headings, keywords, and text words consist of “diabetes” or “diabetes mellitus” combined with “kidney cancer,” or “renal cancer,” or “renal cell carcinoma.” Two reviewers (Chen and Li) independently assessed titles and abstracts of the published papers. No language limitation existed. In addition, references cited in the included studies were reviewed for possible inclusions.

### Study Eligibility

Studies were included if they met the following criteria: cohort studies; studies evaluating the potential association between pre-existing DM and the outcome of RCC; studies that had a median follow-up period of more than 12 months; and studies that reported all-cause mortality, OS, CSS, or recurrence-free survival (RFS) with hazard ratio (HR) and corresponding 95% confidence interval (CI). We also included studies that failed to report 95% CI directly but can be reconstructed to achieve an estimated 95% CI by using *P* values and HR. To avoid incorporating duplicated information, multiple publications from the same author or institution were seriously scrutinized, in which the most informative publication was included. Because the data included in our study were extracted from published literature, ethical approval from ethics committees was not needed.

### Data Extraction

Two investigators (Chen and Gu) independently extracted relevant information from each eligible study using a standardized form. The following items, if available, were extracted: surname of the first author; publication year; origin of the studied population; age of the subjects; sample size; treatment of cancer; follow-up time; and effect estimates, namely, HR of pre-existing DM for OS, CSS, or RFS, as well as their 95% CI and *P* value (recorded or calculated). Disagreements between investigators were resolved through full discussion.

### Quality Assessment

Quality assessment for cohort studies in this meta-analysis was evaluated by using the Newcastle Ottawa Scale (NOS), which was recommended by the Cochrane Non-Randomized Studies Methods Working Group.^16^ Each study was assessed for the following 3 aspects in the scale: selection (total score: 4), comparability (total score: 2), and outcomes (total score: 3). The higher score out of a total of nine points indicated the higher quality, and we considered studies that met 5 or more of the NOS criteria as adequate quality for the meta-analysis.

### Statistical Analysis

Pooled HR with its corresponding 95% CI was calculated to assess the associations of DM with OS, CSS, and RFS of RCC. HR greater than 1 suggested poor prognosis. Statistical heterogeneity for studies reporting the same effect measures was evaluated using Cochrane Q test and Higgins *I*^2^. When no obvious heterogeneity existed among studies (*I*^2^ > 50% suggested high heterogeneity),^17^ the fixed effect model (Mantel–Haenszel method) was used to pool the results. Otherwise, the random effect model (DerSimonian and Laird method) was selected. For additional analyses, meta-analyses were subgrouped on the basis of their clinical stage, pathological type, and therapy (localized RCC, clear cell RCC, surgery). To validate the credibility of outcomes in the meta-analysis and explore the possible explanations for heterogeneity if significant heterogeneity existed, sensitivity analysis was performed by sequential omission of individual studies. Publication bias was evaluated by visual inspection of funnel plots, Begg–Mazumdar adjusted rank correlation test,^18^ and Egger regression asymmetry test.^19^ All analyses were conducted using STATA version 12.0 (State Corporation, College Station, TX, USA). All *P* values were two sided and a *P* value < 0.05 was considered statistically significant.

## RESULTS

### Study Characteristics

**FIGURE 1:**
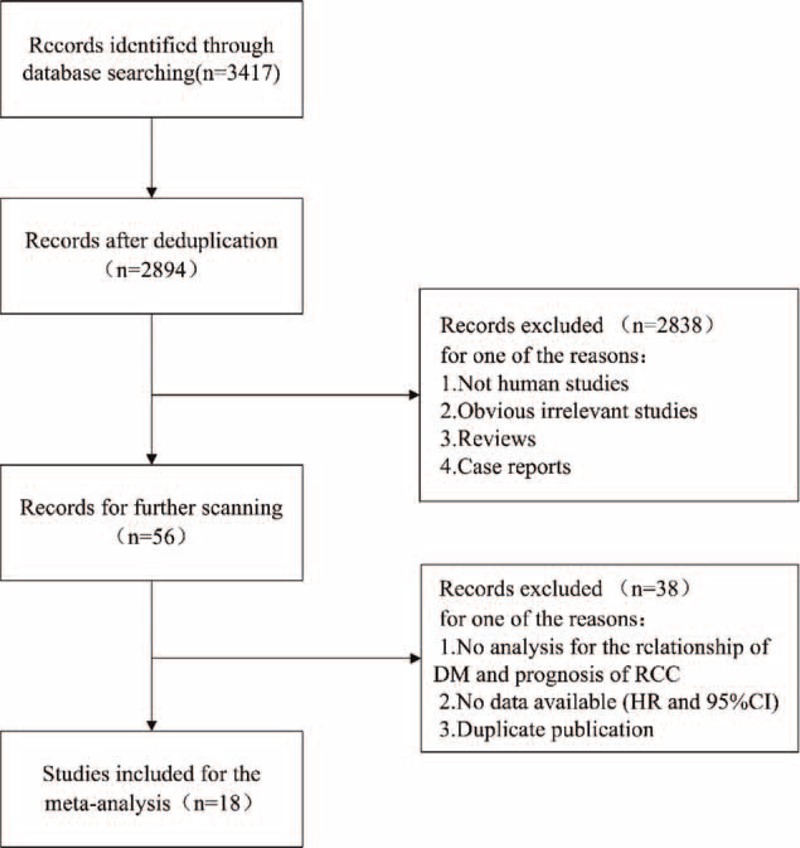
Flow chart of study selection.

A total of 3417 potential relevant studies were identified using a primary literature search in databases. After carefully screening titles and abstracts of identified records, 3361 studies were excluded for reasons such as duplication, animal studies, reviews, case reports, and other apparent irrelevant studies. Of the 56 studies selected for full text assessment, 38 studies that did not refer to the relationship of DM and RCC prognosis, or failed to offer key data (HR and corresponding 95% CI), or belonged to duplicate publication were excluded. Thus, only 18 studies met the criteria for meta-analysis (Figure 1). Table 1 summarizes the descriptive data for the 18 studies. We collected detailed information from these studies including 3 cohort studies that showed only OS or CSS without the exact number of RCC patients. Of the 18 studies, 15 studies^11–13,20–31^ were carried out to investigate OS, 12 studies^11–13,21,24,25,28,29,31–34^ to investigate CSS, and only 3 studies^13,28,29^ referred to RFS, respectively. Patients in these studies were all diagnosed with RCC with or without metastasis from different countries (Korea, Japan, United States, Italy, Germany, Turkey, Israel, and The Netherlands) and received nephrectomy or targeted therapy, with the duration of follow-up of more than 2 years (median time). Moreover, the results of all the studies were adjusted for several confounders, including age, sex, body mass index, obesity, and smoking in the multivariate analyses. Quality scores of the 18 studies ranged from 5 to 9. All were considered adequate for the following meta-analysis.

**TABLE 1:**
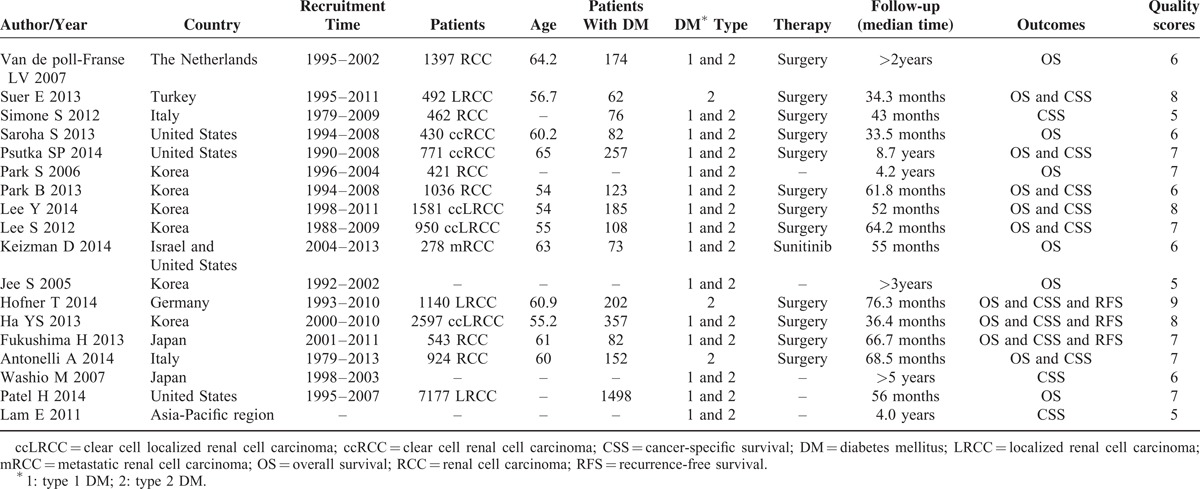
Characteristics of 18 Eligible Studies in the Meta-Analysis

### Meta Analysis

**FIGURE 2:**
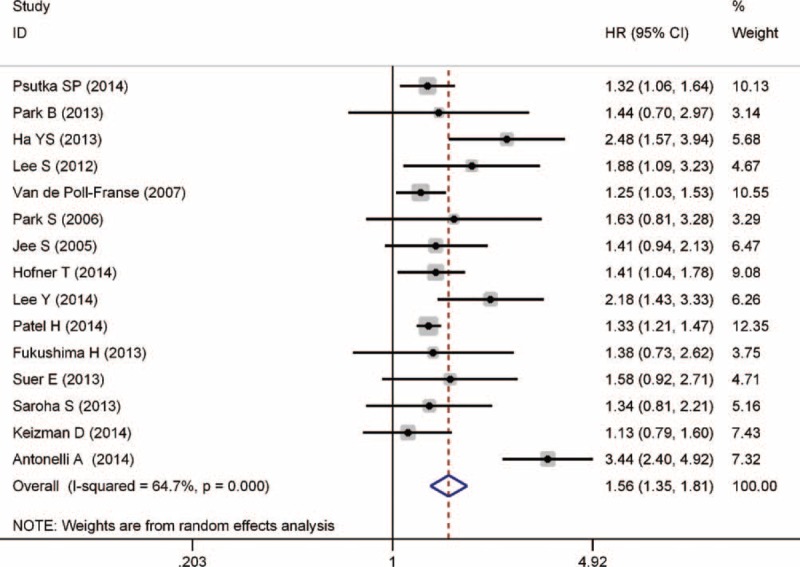
Forest plot of studies evaluating the association between diabetes mellitus and overall survival of renal cell carcinoma.

Of the 15 studies that focused on OS, there was evident interstudy heterogeneity (*P* < 0.001). Thus, a random-effects model was applied to calculate pooled HR and its 95% CI. Figure 2 shows that pre-existing DM significantly predicted worse OS outcome, with pooled HR of 1.56 (95% CI, 1.35–1.81, *P* < 0.001). To validate the credibility of consequence and explore the source of significant heterogeneity, sensitivity analysis by sequential omission of individual studies was performed. This approach did not alter the significance of the combined HR estimate and revealed that the Antonelli 2014 study^31^ is the source of statistical heterogeneity. When this study was removed, there was no significant heterogeneity in the 14 remaining studies (*P* = 0.286, *I*^2^ = 15.3%) and pooled HR of the remaining studies was 1.37 (95% CI, 1.28–1.47, *P* < 0.001). Furthermore, subgroup analyses by clinical stage, pathological type, and therapy suggested that DM was associated with poor OS in localized RCC (*P* < 0.001), clear cell RCC (*P* < 0.001), and RCC with surgical treatment (*P* < 0.001) (Table 2).

**TABLE 2:**
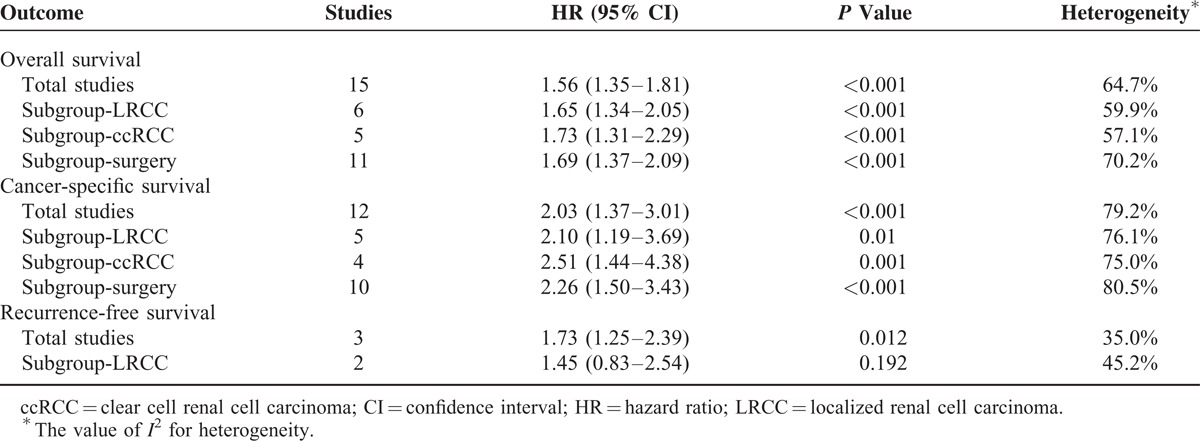
Results of Meta-Analysis of the Association Between Diabetes Mellitus and Prognosis in RCC

**FIGURE 3:**
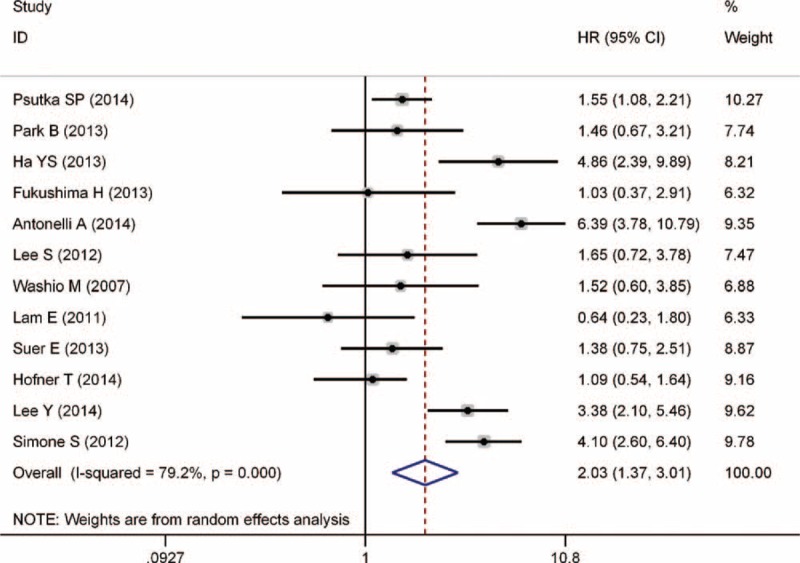
Forest plot of studies evaluating the association between diabetes mellitus and cancer-specific survival of renal cell carcinoma.

Significant heterogeneity between studies that evaluated CSS was apparent (*P* < 0.001). Thus, the random-effects model was used to pool results. The combined HR for CSS was 2.03 (95% CI, 1.37–3.01, *P* < 0.001), indicating that DM was associated with poor CSS in RCC patients (Figure 3). Further sensitivity analysis by sequential omission of individual studies confirmed the credibility of outcomes but did not find the specific reason of significant heterogeneity. Additional subgroup analyses demonstrated that DM was also associated with poor CSS in localized RCC (*P* = 0.01), clear cell RCC (*P* = 0.001), and RCC with surgical treatment (*P* < 0.001) (Table 2).

**FIGURE 4:**
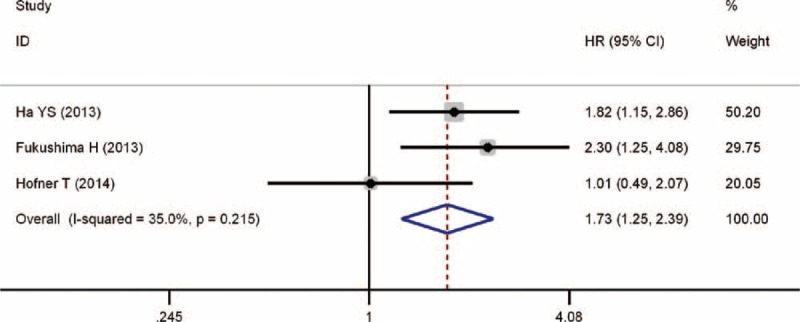
Forest plot of studies evaluating the association between diabetes mellitus and recurrence-free survival of renal cell carcinoma.

Figure 4 shows that 3 studies were eligible for examining the relationship between the DM and RFS of RCC patients. A fixed-effects model was selected because evident heterogeneity among the 3 studies was nonexistent (*P* = 0.215). The pooled HR was 1.73 (95% CI, 1.25–2.39, *P* = 0.012), demonstrating that pre-existing DM had an adverse effect on the RFS of RCC patients who received nephrectomy.

### Publication Bias

**FIGURE 5:**
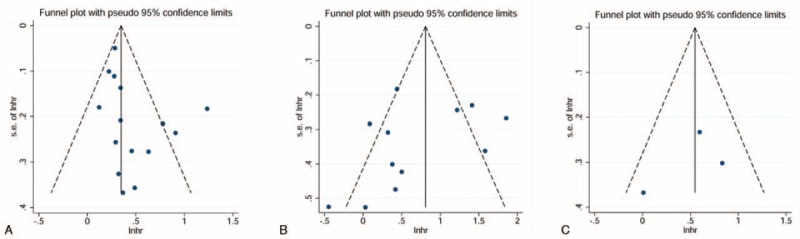
Funnel plots for the evaluation of potential publication bias. A, Overall survival; B, cancer-specific survival; C, recurrence-free survival.

The funnel plot, Egger test, and Begg test were performed to assess the publication bias in meta-analysis. The funnel plots did not reveal obvious evidence of asymmetry in these contrasts (Figure 5). Moreover, the results from the Egger test and Begg test for the studies evaluating OS, CSS, and RFS were *P*_egger's_ = 0.095 and *P*_begg's_ = 0.075, *P*_egger's_ = 0.361 and *P*_begg's_ = 0.451, and *P*_egger's_ = 0.612 and *P*_egger's_ = 1.000, respectively. Thus, the above evidence indicates a low probability of publication bias.

## DISCUSSION

The association between DM and cancer has attracted extensive attention over the recent years. A meta-analysis that included 9 cohort studies by Larsson et al showed that patients with diabetes had a statistically significantly increased risk of kidney cancer compared with individuals without diabetes.^35^ However, previous studies that examined the relationship between DM and kidney cancer prognosis have inconsistent results. Moreover, no precise evidence for the association between DM and survival in RCC patients existed.

In the present research, 18 studies reporting HRs of cumulative survival rates were summarized qualitatively by using standard meta-analysis techniques. Our results indicated a poor OS among kidney cancer patients with DM. Notably, DM also had an adverse effect on CSS, as well as RFS, which is especially important to those who underwent surgically treatment. For patients with localized RCC, patients with clear cell RCC, or patients receiving nephrectomy, DM was also associated with both poor OS and poor CSS by subgroup analyses.

Although several mechanisms have been proposed, the underlying biological linkage between DM and RCC is still largely unknown. It is unclear whether the association is direct or due to share the common metabolic risk factors such as the obesity, which was considered an important factor in cancer development and progression.^36^ Recently, a study by Habib et al suggests that the accumulation of oxidative DNA damage, which was increased through the hyperactivation of Akt/tuberin/mTOR pathway in kidney cancer patients with diabetes, may play an important role in initiating kidney tumorigenesis.^37^ Another convincing mechanism presumed as the effect of hyperglycemia and hyperinsulinemia, which can result in enhanced production of insulin-like growth factor-I (IGF-1) in the liver.^38,39^ Apart from being a potent mediator of tumor cell migration and invasion, IGF-1 is also central in cell proliferation and differentiation.^39^ Increased activation of the IGF-1 signaling pathway has been implicated in many human malignancies including renal cancers.^40^ Moreover, the pathway of IGF-1 and its receptor was identified as a major promoter of tumor invasion and metastases in in vitro and in vivo studies.^41,42^ These above discoveries support our results stating that DM is associated with poor RCC prognosis, and may lead to the proposal that inhibiting the IGF-1 signaling pathway could represent a novel therapeutic strategy.

Compared with the previous study by Bao et al,^43^ our meta-analysis has several strengths. First, in contrast to the previous study, which was conducted in 2012 and included 8 cohort studies focusing on kidney cancer mortality, our meta-analysis included more eligible studies that were published in the recent 2 years with less risk of bias. Second, we applied more stringent selection criteria and did not combine RR and SMR, as in the previous study. Combining RR and SMR with HR was not rational and resulted in an inferior-quality research with a confusing outcome. In addition, subgroup analyses by clinical stage, pathological type, and therapy were performed in our meta-analysis, which were never discussed in the previous study. Finally, besides CSS, we also investigated the OS and RFS, which provided comprehensive evidence for the prognostic role of DM in RCC patients.

Several limitations also exist in the present research. First, a possibility exists that some relevant studies without specific data were not included in this meta-analysis. In addition, pooled HRs could be overestimated as a result of reporting bias that studies with null or nonsignificant results were more difficult to be published than studies with statistically significant results. Second, marked heterogeneity of studies was seen in some analyses (OS and CSS). We attempted to find the exact factor that can account for the heterogeneity by sensitivity analysis. The heterogeneity of OS pooled-analysis may have been due to several design differences among the studies, including patients’ inclusion criteria, which defined only type 2 DM patients without receiving insulin treatment could be included in the Antonelli 2014 study.^31^ Unfortunately, the analyses by current available data did not find evidence of specific contributors to heterogeneity of CSS. Because of the interstudy difference in patients’ characteristics (study size, gender, age, tumor stage and grade, diabetes type), it was difficult to evaluate the precise source of heterogeneity without knowing the original and detailed data of included studies. Besides, several studies that lack complete data might also contribute to part of heterogeneity. Because all included studies were observational in nature, the results might be subject to some unmeasured or residual confounders. In addition, the HRs were adjusted for not identical confounders in multivariate models in different studies, which might also be related to part of heterogeneity. Third, only 3 studies investigated RFS of RCC, which might inevitably increase the risk of random error. In addition, owing to the limited data in the included studies, we cannot analyze the associations between RCC prognosis and type 1 DM or type 2 DM, separately. Moreover, DM duration and therapy were inconsistent or unclear in different studies, which might have an uncertain effect on cancer outcomes. Finally, despite the well-recognized advantages of systematic review and meta-analysis, the most fundamental determinant of quality of synthesized evidence is the quality of the original studies. Therefore, additional well-conducted and appropriately designed studies are needed to demonstrate a more convincing association between DM and RCC.

## CONCLUSION

In summary, our meta-analysis of current evidence suggests that DM is significantly associated with poor OS, CSS, and RFS in RCC patients. More high-quality studies that consider other factors, such as duration and treatment of DM, are needed. Furthermore, more attention should be given to RCC patients with pre-existing DM because of their poor prognosis.
